# Haplotypes in the *CYP2R1* gene are associated with levels of 25(OH)D and bone mineral density, but not with other markers of bone metabolism (MrOS Sweden)

**DOI:** 10.1371/journal.pone.0209268

**Published:** 2018-12-21

**Authors:** Anne Björk, Dan Mellström, Claes Ohlsson, Magnus Karlsson, Hans Mallmin, Gunnar Johansson, Östen Ljunggren, Andreas Kindmark

**Affiliations:** 1 Department of Medical Sciences, Uppsala University, Uppsala, Sweden; 2 Geriatric Medicine, Department of Internal Medicine and Clinical Nutrition, Institute of Medicine, University of Gothenburg, Gothenburg Sweden; 3 Centre for Bone and Arthritis Research at the Sahlgrenska Academy, Institute of Medicine, University of Gothenburg, Gothenburg, Sweden; 4 Department of Clinical Sciences and Orthopedic Surgery, Lund University, Skåne University Hospital, Malmö, Sweden; 5 Department of Surgical Sciences, Uppsala University, Uppsala, Sweden; 6 Department of Public Health and Caring Sciences, Uppsala University, Uppsala, Sweden; Charles P. Darby Children's Research Institute, 173 Ashley Avenue, Charleston, SC 29425, USA, UNITED STATES

## Abstract

**Objective:**

Polymorphisms in the *CYP2R1* gene encoding Vitamin D 25-hydroxylase have been reported to correlate with circulating levels of 25-OH vitamin D3 (25(OH)D). It is unknown whether these variations also affect overall bone metabolism. In order to elucidate the overall associations of polymorphisms in the *CYP2R1*, we studied haplotype tagging single nucleotide polymorphisms (SNPs) in the gene and serum levels of 25(OH)D, calcium, phosphate, parathyroid hormone (PTH) and fibroblast growth factor-23 (FGF23), as well as bone mineral density (BMD).

**Methods:**

Baseline data on serum parameters and BMD from MrOS Sweden, a prospective population-based cohort study of elderly men (mean age 75 years, range 69–81), were analyzed. Genotyping was performed for eight SNPs covering the *CYP2R1* gene in 2868 men with available samples of DNA. Subjects were followed up concerning incidence of fracture during five years.

**Results:**

There was a significant genetic association with circulating levels of 25(OH)D (4.6–18.5% difference in mean values between SNP alleles), but there were no correlations with levels of calcium, phosphate, PTH or FGF23 for any genetic variant. No differences were found in fracture incidence between the variants. There was an inverse relationship between lower BMD and concomitant higher 25(OH)D for three of the haplotypes (p < 0.005).

**Conclusions:**

Common variants in the *CYP2R1* gene encoding Vitamin D 25-hydroxylase correlate with levels of circulating 25(OH)D but do not otherwise associate with measures of calcium and phosphate homeostasis. Presence of the specific haplotypes may be an indicator of risk for low 25(OH)D levels, and may in addition be correlated to bone mineral density.

## Introduction

The increasing incidence of osteoporosis-related fractures with increasing age is a major health problem, leading to suffering and increased mortality, as well as economic problems for both the individual and society [1, 2]. Osteoporosis is characterised by low bone mineral density (BMD), and bone micro architectural deterioration. Both environmental and hereditary factors have been shown be important and to interact, for BMD as well as for fractures [3–5].

Twin and family-based osteoporosis studies have indicated that as much as 60 to 85% of the variance in BMD is genetically determined [6], and genome-wide association studies (GWAS) have identified single nucleotide polymorphisms (SNPs), associated with low BMD, osteoporosis and osteoporotic fractures [7].

Calcium (Ca), phosphate (P), and vitamin D are all essential for bone metabolism and the maintenance of the strength and function of the skeleton. The role of vitamin D, in this context, is to participate in the regulation of Ca homeostasis. Thus, the active metabolite of vitamin D stimulates calcium absorption from the gut. Parathyroid hormone (PTH) acts on the skeleton by enhancing the release of calcium from the bones. It also regulates renal calcium handling. By stimulating the conversion of 25(OH)D into the active form 1,25-dihydroxyvitamin D3, PTH also causes an enhanced absorption of calcium in the intestine. Another important phosphate regulating factor participating in bone and mineral metabolism is the fibroblast growth factor 23 (FGF23) which in turn is regulated by levels of phosphate and 1,25-dihydroxyvitamin D3 [8, 9]. Vitamin D deficiency can lead to musculoskeletal diseases such as rickets and osteomalacia, but vitamin D supplementation may also prevent extra skeletal diseases such as respiratory tract infections, asthma exacerbations, pregnancy complications and premature deaths. [10]

Vitamin D deficiency leads to a decrease in intestinal calcium absorption and ultimately to a transient decrease in ionized calcium. The 25(OH)D-concentration in blood is regarded as the best measure of an individual’s overall vitamin D status [11].

The diet may contain vitamin D2 (ergocalciferol) or vitamin D3 (cholecalciferol), the latter mainly in fish, dairy products or additions to margarine or milk. Most supplements contain cholecalciferol. Ergocalciferol is generated in some foods (mushrooms) by UV radiation. Whether produced in the body from the diet (mainly in fish, dairy products, additions to margarine or milk and supplements) or dermal synthesis from sunlight containing UVB, vitamin D is initially biologically inactive, and activation requires enzymatic conversion (hydroxylation) in the liver and kidney. The enzyme vitamin D 25-hydroxylase, encoded by the *CYP2R1* gene, has been shown to be a key enzyme for the conversion in the liver of cholecalciferol into the form (calcidiol) [12]. Variants near the *CYP2R1* gene have also been shown to influence circulating levels of 25(OH)D, and genome-wide significance for association between levels of the vitamin has been shown for one of the *CYP2R1* SNPs; rs10741657 [13]. A Danish study has also shown that common polymorphisms in the vitamin D binding protein (VDBP encoded by the gene GC) and *CYP2R1* are associated with 25(OH)D concentrations in the Caucasian population and that certain haplotypes may predispose to lower 25(OH)D concentrations in late summer in Denmark [14].

Genetic variation is in turn also believed to explain 79.4% of the variation of levels of the VDBP, but only 9.9% of the variation in 25(OH)D levels [15]. Several studies indicate that allelic variation in *CYP2R1* (rs10741657) affects vitamin D levels [16–18].

It is not clear how other biochemical markers and BMD are associated with polymorphisms in the *CYP2R1* gene. Apart from a study of postmenopausal Chinese women which showed no significant association between 10766197 and BMD, no genetic association studies analyzing the *CYP2R1* gene and BMD have been published [19].

The aims of the present study were to investigate the relationship between polymorphisms and haplotypes in the *CYP2R1* gene and levels of 25(OH)D, as well as other biochemical parameters (PTH, Ca, P and FGF23) of calcium homeostasis. In addition, possible associations between genetic variation in *CYP2R1* with BMD of the hip, lumbar spine and femoral neck as well as fracture incidence during the five years following baseline were investigated.

## Materials and methods

### Subjects

The MrOS study is a multi-center, prospective cohort study of elderly men in Sweden, Hong Kong and the USA [20]. The present study used data from the Swedish part of MrOS (n = 3014), recruited at medical centers in Uppsala (n = 999), Gothenburg (n = 1010) and Malmö (n = 1005). Men aged 69–81 years were randomly identified using national population registers. To be eligible for the study, participants had to be able to walk without aids, provide self-reported data and give signed informed consent. There were no other exclusion criteria. The participation rate in MrOS Sweden was 45%. In the present report, the baseline data in MrOS Sweden were used for biochemical markers and BMD measurements. Fracture data available after 5 years from baseline were analyzed. Diabetes incidence was estimated from a health questionnaire at baseline., The diagnoses were not validated by searching in the charts.

Informed consent was obtained for all subjects and the study was approved by the l The Regional Ethical Review Board in Lund, (Dnr LU 693–00), the Central Ethical Review Board at Gothenburg university (Dnr Gbg M 014–01) and the Regional Ethical Review Board in Uppsala (Ups 01–057). The study was performed in accordance with the declaration of Helsinki.

### Genotyping of the CYP2R1 gene

DNA was isolated from whole blood extracted at baseline from all participants where blood samples were available, in total 2870 participants. Using a saturation approach representing HapMap SNPs and HaploView scoring, a total of 11 SNPs covering 100 kb of the genetic region surrounding the *CYP2R1* gene including the 3’ and 5’ untranslated regions (UTRs) were selected. Genotyping was performed using the Sequenom Mass ARRAY iPLEX Gold technology (Sequenom Inc., Newton, MA) by single base primer extension and MALDI TOF Mass Spectrometry. Successful genotyping was obtained from 8 SNPs (rs10766197, rs11023374, rs10741657, rs10832313, rs16930609, rs16930625, rs11023371 and rs7936142) with overall call rate of 97.8%. Allele frequencies were calculated and found to be in Hardy-Weinberg (HW) equilibrium in the cohort for all SNPs. Haploview software version 4.2 was used to calculate linkage disequilibrium (LD) values, generate haplotype blocks and diagrams, as well as suggesting tagging SNPs using the tagger algorithm [21]. The preselected SNPs and haplotypes computed using the Arlequine population genetic data analysis program, were analyzed for associations between vitamin D values and other biochemical parameters (calcium, phosphate, FGF-23 and PTH), as well as markers of bone mineral density (femoral neck, lumbar spine and total hip).

### Serum measurements

Serum samples were collected and stored at -80°C for biochemical markers, and at -20°C for DNA analysis.

Serum 25(OH)D was measured at baseline in 2878 subjects, with a competitive RIA (Diasorin, Stillwater, MN, USA; intra-assay CV 6%, inter-assay CV 15–16%) at a single laboratory. The inter-assay CV was 15–16% at all 25(OH)D levels [22]. The laboratory used participated in DEQAS quality controls.

Phosphate, calcium and albumin were analyzed at respective hospitals department for clinical chemistry using standard methods. Albumin modified calcium was calculated with the formula calcium-(0.018(albumin-42)) [23]. Estimated glomerular filtration rate (eGFR) in ml/min/1.73 m^2^ was calculated from serum cystatin C (Cystatin C Immunoparticles, Dako A/S, Glostrup, Denmark) according to the formula 79.901*(Cyst C [mg/L])^-1.4389^ [24]. Intact PTH was measured by a second generation immunometric assay, Immulite 2000, (Los Angeles, USA). 25(OH)D levels were measured by Nichols Advantage automated assay system (San Juan Capistrano, CA, USA). Serum concentration of intact FGF23 was analyzed in using a two-site monoclonal antibody-based ELISA (Kainos Laboratories International; Tokyo, Japan).

### BMD measurements

BMD of the lumbar spine, total hip and femoral neck was measured using DXA scanners: Lunar Prodigy DXA (GE Lunar Corp., Madison, WI, USA) in Malmö and Uppsala and Hologic QDR 4500/ A-Delphi (Hologic, Bedford, MA, USA) in Gothenburg. DXA measurements performed with equipment from different manufacturers were converted to a standardized BMD as previously described [25–27].

### Fracture data

Study participants were followed up for a mean of 5.9 years (range 4.7–7.4) after the baseline examination. They received a one-page Tri-Annual Questionnaire every four months. This instrument was used to update contact information and to ascertain the incidence of falls and fractures and back pain. Time to first fracture or death was defined as time from the baseline study date to the actual event. Fracture evaluation during follow-up was in addition done by re-evaluation of X-ray in the regional registry, identified by the probands' unique personal registration number[20, 28].

The following fractures were regarded as osteoporotic: fractures of the pelvis, vertebrae, radius and humerus.

### Other measurements

Height (in centimeters) and weight (in kilograms) were measured, and BMI was calculated as kilograms per square meter.

### Statistical analysis

Statistical analysis was performed using the IBM SPSS program version 22 and SAS version 9.4. Differences between characteristics for the different SNPs were computed by ANOVA and Tukey’s post hoc testing. p<0.05 were considered significant. Values are given as mean ± SD unless otherwise stated. Probability for deviation from Hardy-Weinberg equilibrium (HWE), and major and minor allele frequencies were calculated using χ^2^ test for HW equilibrium for biallelic markers. Differences in relative fracture risk between alleles of tagging SNPs were compared by calculating chi-square. The analyses were done for osteoporotic fractures in all participants with data on genotype and fracture. No adjustments for covariates were made.

## Results

### Subjects

Genotyping and serum 25(OH)D concentrations were available for 2870 participants. The mean age was 75.4 years (range 69–81), and mean BMI was 26.4. Height, weight and Body Mass Index (BMI) were all normally distributed. Overall, the participants were relatively vitamin D sufficient, and the mean level of 25(OH)D was 69.8 nmol/L. Only 0.9% had vitamin D deficiency (<25 nmol/L), and 17% had vitamin D insufficiency (25–49 nmol/L). The incidence of self-reported diabetes was 9.5%.

Characteristics of the study cohort, biochemical parameters and BMD are summarized in Table 1.

**Table 1:** Description of the study cohort (N = 2870).

Characteristics	Mean	SD
**Age (years)**	75.4	3.2
**Height (m)**	1.75	0.07
**Weight (kg)**	80.8	12.1
**BMI (kg/m**^**2)**^	26.4	3.6
**Current smokers N (%)**	241 (8.4)	
**25(OH)D (nmol/L)**	69.8	23.8
**Albumin correlated calcium (mmol/L)**	2.34	0.16
**Phosphate (mmol/L)**	1.07	0.16
**PTH (pmol/L)**	4.64	3.0
**FGF23 (pg/mL)**	48.6	37.8
**Albumin (g/L)**	43.1	3.6
**Cystatin C (mg/L)**	1.14	0.30
**Estimated GFR (ml/min/1.73 m**^**2)**^	72.0	20.6
**Lumbar spine vertebra 1–4. standardized BMD (mg/cm**^**2**^**)**	1142.8	202.2
**Total hip, standardized BMD (mg/cm**^**2**^**), left hip**	948.7	145.7
**Femoral neck, standardized BMD (mg/cm**^**2**^**), left hip**	840.3	132.7

Demographic data, biochemical parameters and bone mineral density at baseline.

Continuous data are shown as means with SD, and categorical data as numbers (percentages).

BMI = Body Mass Index, PTH = Parathyroid hormone, FGF23 = Fibroblast growth factor 23, GFR = Glomerular filtration rate, BMD = Bone Mineral Density.

### Haplotype analysis

**Fig 1:**
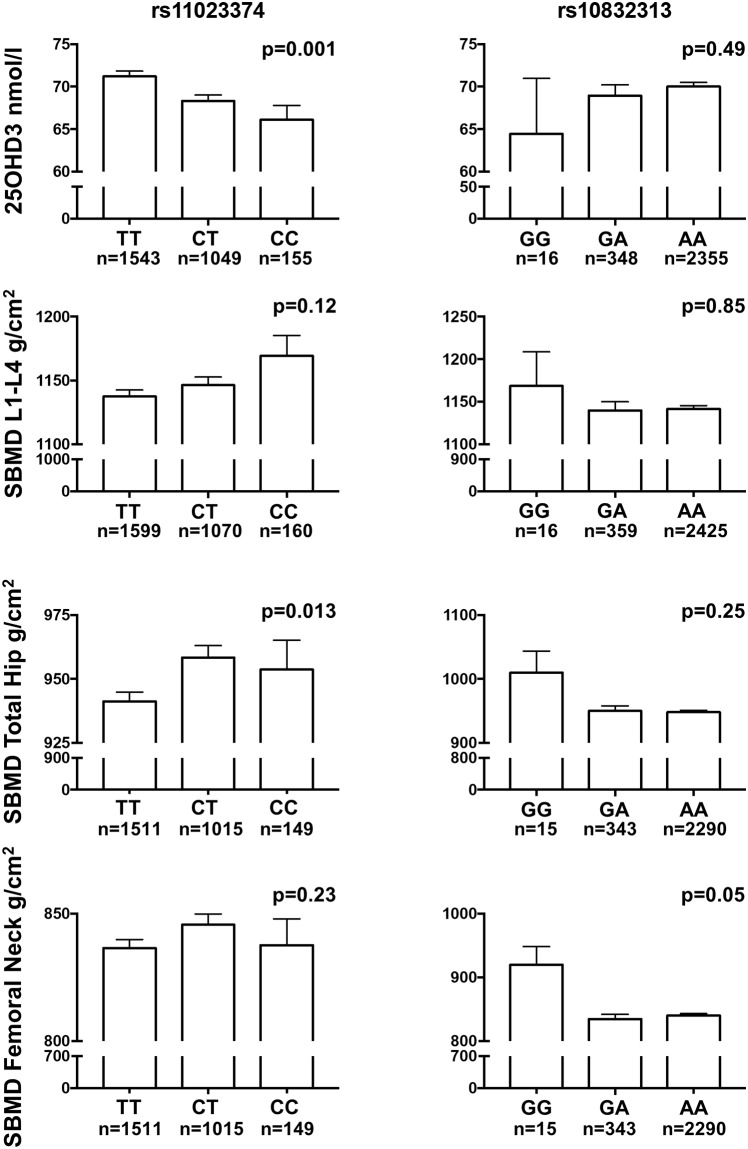
Genetic structure of the *CYP2R1* gene, with Linkage Disequilibrium (LD) plot for the eight analysed SNPs and definition of the six most common haplotypes of the *CYP2R1* gene. The location of each SNP is indicated on top and the number in each diamond indicates the magnitude of LD between respective pairs of SNPs. Empty squares represent perfect LD. The table below the diagram shows the SNP genotype combination defining the 6 most common haplotypes (1–6).

The eight preselected SNPs covered the regulatory region and the exonic and intronic regions of the *CYP2R1* gene. Presence of the 6 most common haplotypes was found in 93.6% of the subjects (Fig 1).

### Laboratory markers

1. 25 OHD: Statistical analysis with ANOVA showed significant association (p < 0.05) between on one hand each of the haplotypes 1, 2, or 6, and on the other hand 25(OH)D levels. Analysis of all homozygotes and heterozygotes for these haplotypes showed higher 25(OH)D levels for homozygotes of haplotypes 1 and 2, but significantly lower levels for homozygotes or heterozygotes which included haplotype 6 (Table 2).
2. Other biochemical parameters: Analysis with ANOVA of the other biochemical parameters of calcium and phosphate homeostasis (PTH, Ca, FGF-23 and phosphate) showed no associations with regard to haplotype combination. (Table 2).

**Table 2:** Descriptive statistics: 25(OH)D-levels and BMD for the six most common haplotypes.

Haplotype	N	Numbers of copies of haplotype					BMD (g/cm^3^)			
			25(OH)D (nmol/L)	p-value^a^	Lumbar spine	p-value	Total hip	p-value^a^	Femoral neck	p-value^a^
**1**	1636	0	71.1 (24.7)	**0.001**	1139.4 (200.2)	0.296	942.0 (143.1)		836.8 (131.4)	0.179
	988	1	68.2 (22.6)		1145.0 (207.0)		959.5 (150.1)	**0.013**	846.7 (135.6)	
	142	2	65.6 (20.3)		1165.7 (190.2)		952.2 (141.0)		836.7 (125.6)	
**2**	1636	0	69.4 (23.1)	**0.002**	1143.4 (202.4)	0.733	947.1 (144.4)		838.1 (132.0)	0.603
	988	1	69.1 (23.9)		1140.0 (198.3)		949.5 (143.8)	0.762	841.6 (130.0)	
	142	2	74.6 (26.5)		1150.4 (216.3)		953.9 (159.4)		846.4 (146.5	
**3**	2340	0	69.5 (23.6)	0.150	1145.1 (201.9)	0.361	950.6 (146.3)		841.7 (133.2)	0.322
	412	1	71.7 (25.1)		1129.9 (205.2)		938.3 (142.6)	0.272	833.0 (130.4)	
	14	2	74.2 (22.6)		1142.4 (142.7)		932.9 (133.7)		808.0 (104.9)	
**4**	2071	0	70.0 (24.1)	0.617	1145.0 (202.5)	**0.038**	951.7 (146.6)		842.1 (133.7)	0.142
	628	1	68.9 (22.6)		1141.7 (200.2)		943.3 (142.9)	**0.025**	837.4 (129.4)	
	87	2	69.3 (25.1)		1080.6 (204.8)		905.8 (136.5)		810.8 (128.9)	
**5**	2476	0	70.1 (23.8)	0.086	1143.8 (202.8)	0.663	948.8 (145.6)		841.1 (132.3)	0.068
	278	1	67.3 (23.9)		1133.2 (197.8)		944.8 (146.7)	0.305	829.9 (136.5)	
	12	2	61.4 (19.9)		1163.0 (167.9)		1013.5 (135.9)		916.0 (99.3)	
**6**	2345	0	69.2 (23.9)	**0.005**	1144.2 (202.9)	0.353	949.5 (145.9)		840.2 (131.9)	0.988
	401	1	72.1 (23.1)		1137.2 (199.2)		944.6 (145.5)	0.783	840.7 (138.5)	
	20	2	82.3 (23.8)		1086.0 (165.8)		937.7 (131.5)		836.1 (112.5)	

25(OH)D, 25OH vitamin D; BMD bone mineral density. Mean values and standard deviations. N = 2870.

^a^p-values were calculated using ANOVA.

#### BMD

Multiple linear regression, with outcomes lumbar spine BMD, total hip BMD, femoral neck BMD analysed separately for each of the SNPs and HAPs, adjusted for age, showed significant associations (p<0.05) for the SNPs rs11023374 with total hip BMD and rs10832313 with femoral neck BMD, haplotype 4 with lumbar spine BMD and total hip BMD, and haplotype 1 with total hip BMD. (Table 3).

**Table 3:** Analysis of associations between SNPs/haplotype and bone mineral density, adjusted for age. Outcomes: lumbar spine BMD, total hip BMD, femoral neck BMD analysed separately for each of the SNPs and HAPs.

				Lumbar spine BMD		Total Hip BMD		Femoral neck BMD	
SNP / HAP	Level	N	%	p-value^a^	beta estimate	p-value^a^	beta estimate	p-value^a^	beta estimate
**rs10766197**	TT (ref)	518	18.53	0.9774		0.9417		0.9494	
	CC	898	32.12						
	CT	1380	49.36						
**rs11023374**	GA (ref)	1065	37.74	0.1069		**0.012**		0.2538	
	A	1598	56.63			0.0035	-17.287756		
	G	159	5.63			0.7821	-3.538543		
**rs10741657**	TT (ref)	560	19.64	0.7742		0.8243		0.9558	
	CC	922	32.34						
	CT	1369	48.02						
**rs10832313**	TT (ref)	2440	86.59	0.8594		0.2585		**0.0437**	
	CC	16	0.57					0.0207	78.891876
	CT	362	12.85					0.3768	-6.742963
**rs16930609**	TT (ref)	2358	82.33	0.1847		0.7073		0.8131	
	GG	26	0.91						
	GT	480	16.76						
**rs16930625**	TT (ref)	2369	82.75	0.6178		0.876		0.9887	
	CC	28	0.98						
	CT	466	16.28						
**rs11023371**	CC (ref)	2311	81.23	0.3371		0.2825		0.3812	
	CT	512	18						
	TT	22	0.77						
**rs76936142**	TT (ref)	2494	87.69	0.8316		0.3868		0.1272	
	AA	15	0.53						
	TA	335	11.78						
**Haplotype 1**	0 (ref)	1709	59.55	0.2948		**0.0106**		0.1594	
	1	1014	35.33			0.0027	17.749128		
	2	147	5.12			0.4204	10.401654		
**Haplotype 2**	0 (ref)	1473	51.32	0.737		0.8108		0.6514	
	1	1116	38.89						
	2	281	9.79						
**Haplotype 3**	0 (ref)	2429	84.63	0.3584		0.1992		0.2225	
	1	427	14.88						
	2	14	0.49						
**Haplotype 4**	0 (ref)	2147	74.81	**0.0387**		**0.0406**		0.2069	
	1	654	22.79	0.7149	-3.313495	0.2457	-7.736133		
	2	69	2.4	0.0109	-64.322702	0.0192	-42.694266		
**Haplotype 5**	0 (ref)	2568	89.48	0.6623		0.269		0.0569	
	1	290	10.1						
	2	12	0.42						
**Haplotype 6**	0 (ref)	2437	84.91	0.3552		0.8161		0.9953	
	1	412	14.36						
	2	21	0.73						

*P-values were calculated using multiple linear regression, adjusted for age. Type 3 p-values are displayed showing the strength of association between the outcome variable and the SNP/Hap. If Type 3 p-value was significant (<0.05), Pr>|t| are displayed to show the significance of difference to the reference level.

When subjects with self-reported diabetes (N = 274 were removed, significant associations were found between 25(OH)D and 4 of the haplotypes (1,2,3 or 6). Significant associations were still seen between haplotype 4 and BMD (lumbar spine and total hip) in non-diabetic subjects (p < 0.05).

**Fig 2:**
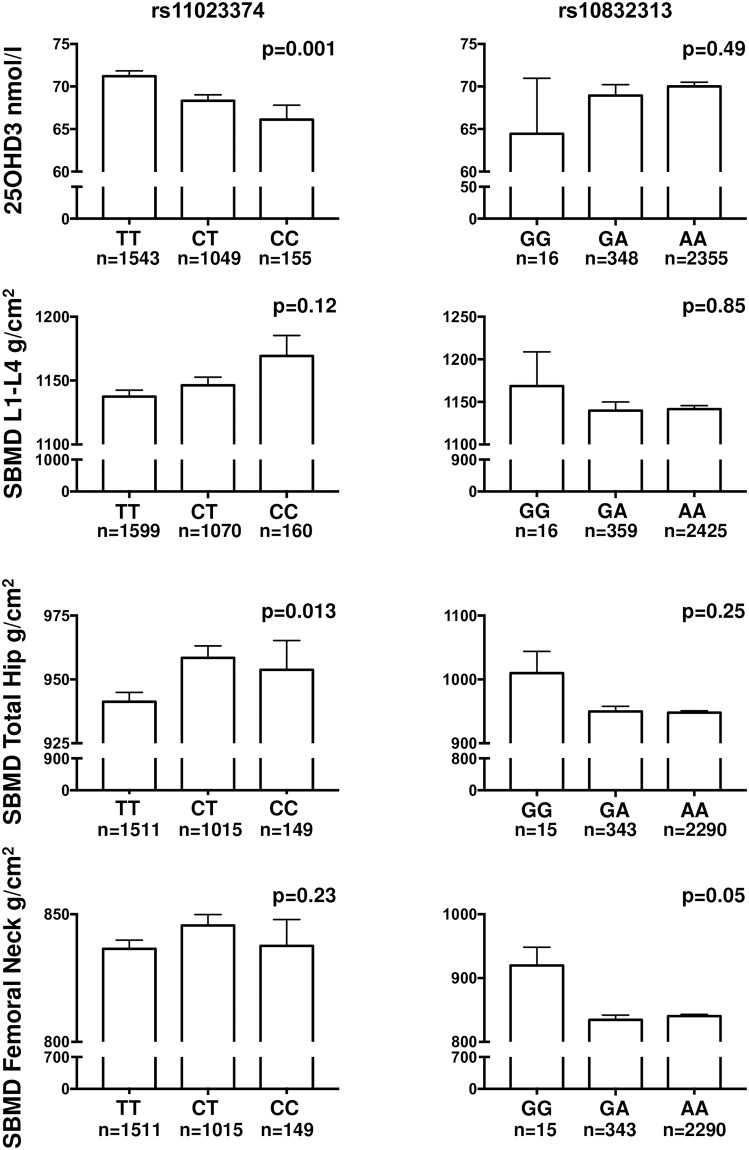
Variation in 25(OH)D concentrations and standardized BMD (SBMD) of the lumbar spine (L1-L4), total hip and femoral neck by *CYP2R1* SNPs rs11023374 and rs10832313 genotypes. Similar, although not significant, patterns (i.e. low 25(OH)D levels were associated with higher BMD) were seen for all SNPs. Values are presented as mean and SEM, with overall p-values by ANOVA.

When analysing the SNPs separately by ANOVA, six of the eight SNPs were significantly associated with circulating levels of 25(OH)D (4.6–18.5% difference in mean values between SNP genotypes), but no correlations with circulating levels of calcium, phosphate, PTH or FGF23 for any of the SNPs were found. There was a slightly higher BMD (0.07–6.5% in the lumbar spine and 5.1–6.9% in the femoral neck) for two SNPs variants (rs11023374 and rs10832313), associated with lower circulating 25(OH)D levels. Interestingly, the higher BMD of the total hip (p = 0.013) found for rs11023374 variants was associated with lower 25(OH)D levels (Fig 2). Furthermore, a higher BMD of the femoral neck (p = 0.05) was seen for the CC allele of rs10832313. For the other SNPs, no significant differences in BMD were found between SNP genotypes, but there was a clear trend for all SNPs that lower 25(OH)D levels were associated with higher BMD values (Fig 2).

### Fracture incidence

The overall incidence of osteoporotic fractures in the cohort up to five years of follow up was 438 (15.3%) (Table 4), No significant differences were found in osteoporotic fracture incidence between variants of *CYP2R1* for any of the haplotypes nor SNPs.

**Table 4:** Incidence of osteoporotic fractures. N = 2870.

Localisation	Frequency	Percent
Acetabulum	5	0.2
Radius	46	1.6
Lumbar vertebra	78	2.7
Neck of femur	80	2.8
Pubis	16	0.6
Thoracic vertebra	90	3.1
Humerus	37	1.3
Tibia	4	0.1
Thoracic spine	22	0.8
Pertrochanteric	54	1.9
Subtrochanteric	6	0.2
Total	438	15.3

## Discussion

Our results show that genetic variation in six of the eight SNPs covering the *CYP2R1* gene were associated to serum 25(OH)D levels but not to other markers for calcium-phosphate balance. These findings with respect to 25(OH)D levels are consistent with other studies on subjects of European ancestry [3, 13, 16], including a Danish study, showing that alleles GG/AA of the SNPS rs19741657 and rs10766197 haplotypes were related to lower 25(OH)D concentrations [14]. Our results are, however, contrary to studies of Chinese women where no significant associations were found. This could possibly be explained by gender or ethnic differences [29].

One might expect that the genetic variants associated with low vitamin D levels would be associated with elevated PTH levels, since vitamin D deficiency often causes secondary hyperparathyroidism, with elevated PTH levels. However, although *CYP2R1* haplotype number 6 (GTAGCGGA) was found to be predictive of lower 25(OH)D concentrations in our study cohort, neither this haplotype nor any other variant was associated with levels of the other markers of calcium and phosphate homeostasis (Ca, PTH, FGF23 and phosphate), nor with bone mineral density. A possible explanation of this could be that these parameters are controlled by other mechanisms than only the levels of 25(OH)D. As to the other parameters of calcium and phosphate homeostasis, no previous studies have been published, according to our knowledge.

An association was seen for one SNP (rs11023374) to BMD of the hip, and similar (although not significant) trends were seen for all the other SNPs for BMD in femoral neck and lumbar spine. Intriguingly, our results show that the SNP variants associated to low 25(OH)D levels were associated with higher BMD and vice versa, the tendency seen for all SNPs. Mechanistically, it could be possible that the action is conferred on the bone cellular level with local vitamin D conversion depending on the *CYP2R1* genotype, rather than an effect on circulating levels of 25(OH)D. There have been no previous reports on association between *CYP2R1* genetic variants and BMD or fractures. In a study of a cohort of 342 subjects in Austria, no association between the rs10741657 SNP and 5 years follow up of fracture incidence was found [3].

A strength of our study is that the cohort is one of the largest available male study cohorts in the world, homogenous with respect to genetic background, and that a similar pattern with regard to 25(OH)D and BMD was seen for several SNPs. A weakness of the study is that although the association between *CYP2R1* variants and BMD is clear, a mechanistic explanation is currently lacking. Also, in this cohort of elderly Swedish men, presence of clinical vitamin D deficiency was rare, and these results might have been different for another population, with higher incidence of low 25(OH)D levels in the blood. Furthermore, our study does not include measurements of vitamin D binding protein.

## Conclusions

This study demonstrated that genetic variants of the *CYP2R1* gene were correlated to levels of circulating 25(OH)D, but not to calcium, phosphate, PTH, nor FGF-23. The genetic variant associated with a concomitant inverse relationship between 25(OH)D and BMD needs further investigation. Presence of the one *CYP2R1*haplotype (GTAGCGGA) appears to be an indicator of risk for low 25(OH)D levels, but it remains to elucidate whether there could be any risks of severe deficiency.

## Supporting information

S1 Table Associations between serum parameters for 8 different SNPs in *CYP2R1*, presenting p-values by ANOVA. **N = 2870**. SNP = Single Nucleotide Polymorphism, PTH = Parathyroid hormone, FGF23 = Fibroblast growth factor 23, GFR = Glomerular filtration rate.
(DOCX)
