# Supplementary material for: Haplotypes in the CYP2R1 gene are associated with levels of 25(OH)D and bone mineral density, but not with other markers of bone metabolism (MrOS Sweden)
Source: PLoS One. 2018 Dec 21;13(12):e0209268. doi: 10.1371/journal.pone.0209268 (PMC6303094; doi:10.1371/journal.pone.0209268)
Supplement: S1 Table — N = 2870. SNP = Single Nucleotide Polymorphism, PTH = Parathyroid hormone, FGF23 = Fibroblast growth factor 23, GFR = Glomerular filtration rate. (DOCX) [file pone.0209268.s001.docx]

**Supplementary table. Associations between serum parameters for 8 different SNPs in *CYP2R1*, presenting p-values by ANOVA. N = 2870.**

| SNP | 25(OH)D | Albumin correlated calcium | Phosphate | PTH | FGF23 | Estimated GFR |
| --- | --- | --- | --- | --- | --- | --- |
| rs11023371 | 0.45 | 0.82 | 0.74 | 0.99 | 0.27 | 0.49 |
| rs11023374 | 0.001 | 0.53 | 0.27 | 0.84 | 0.88 | 0.79 |
| rs7936142 | 0.01 | 0.08 | 0.74 | 0.49 | 0.58 | 0.84 |
| rs10741657 | 0.005 | 0.59 | 0.37 | 0.52 | 0.56 | 0.41 |
| rs16930609 | 0.005 | 0.60 | 0.61 | 0.75 | 0.48 | 0.93 |
| rs16930625 | 0.02 | 0.22 | 0.84 | 0.57 | 0.68 | 0.84 |
| rs10766197 | 0.001 | 0.18 | 0.24 | 0.85 | 0.32 | 0.47 |
| rs10832313 | 0.49 | 0.22 | 0.85 | 0.53 | 0.44 | 0.88 |

SNP=Single Nucleotide Polymorphism, PTH=Parathyroid hormone, FGF23=Fibroblast growth factor 23, GFR=Glomerular filtration rate
